# Citrus Polyphenols in Brain Health and Disease: Current Perspectives

**DOI:** 10.3389/fnins.2021.640648

**Published:** 2021-02-19

**Authors:** Matthew G. Pontifex, Mohammad M. A. H. Malik, Emily Connell, Michael Müller, David Vauzour

**Affiliations:** Norwich Medical School, Biomedical Research Centre, Faculty of Medicine and Health Sciences, University of East Anglia, Norwich, United Kingdom

**Keywords:** flavonoids, orange juice (OJ), cognition, gut-brain axis, neuroprotection

## Abstract

In addition to essential micronutrients such as vitamin C, citrus fruits represent a considerably rich source of non-essential bioactive compounds, in particular flavanones which form a sub-set of the flavonoid group. Preclinical studies have demonstrated the neuroprotective potential of citrus flavonoids and have highlighted both the well-established (anti-inflammatory and anti-oxidative properties), and newly emerging (influence upon blood-brain barrier function/integrity) mechanistic actions by which these neurological effects are mediated. Encouragingly, results from human studies, although limited in number, appear to support this preclinical basis, with improvements in cognitive performance and disease risk observed across healthy and disease states. Therefore, citrus fruits – both as whole fruit and 100% juices – should be encouraged within the diet for their potential neurological benefit. In addition, there should be further exploration of citrus polyphenols to establish therapeutic efficacy, particularly in the context of well-designed human interventions.

## Introduction

A varied and balanced diet, rich in plant-derived material (fruit and vegetables), is widely regarded as optimal for maintaining overall health and wellbeing. Unfortunately, typical modern dietary preferences are increasingly skewed in favor of nutrient poor, calorie dense, ultra-processed food choices. Combined with the background of an aging population, circumstances have produced a ‘perfect storm’ leading to a surge in metabolic disease burden, including rising incidence of neurodegenerative conditions which present significant societal and economic challenges.

Plant bioactives such as polyphenolic compounds have shown potential in negating the metabolic disease process (Chiva-Blanch and Badimon, 2017). Polyphenol describes a broad range of non-essential, usually naturally occurring compounds of which fruits and vegetables represent a significant source. The use of polyphenolic compounds in a therapeutic capacity is being increasingly considered, as we ‘return to nature’ to find sources of potential drug candidates (Xiao, 2017; Bellavite and Donzelli, 2020). Although currently lacking confirmation through rigorous long-term randomized control trial (RCT) evidence, the use of polyphenolic compounds in the prevention of cardiovascular disease appears promising (Parmenter et al., 2020). Indeed, an inverse relationship between polyphenolic compound consumption and cardiovascular disease mortality, risk and biomarkers are consistently highlighted in observational and preclinical studies. Given that cardiovascular health is a contributing factor to brain health, and cardiovascular disease is a significant risk factor for various forms of dementia, the benefits of polyphenols may also extend to brain functions.

Intriguingly, the mechanistic basis by which polyphenols may contribute to brain health and cognition extends further than those associated with cardiovascular health to include anti-inflammatory and anti-oxidative capabilities (Vauzour et al., 2017; Flanagan et al., 2020). In addition, the developing role of the gut microbiota and its interplay with plant bioactives offers a novel route by which physiological effects can be exerted (Kumar Singh et al., 2019; Catalkaya et al., 2020). Continuing to reveal the underlying biochemical processes to which these compounds exert their effects is essential to utilizing their potential in brain health and disease, however, the importance of evaluating these compounds clinically cannot be underestimated. In this review, we will examine the current evidence on polyphenols and their effects on brain health, focusing specifically on citrus polyphenols since these are one of the main groups of flavonoids in the European diet.

## Plant Polyphenols

Synthesized by plants to form either structural/functional tissue, or as a defense mechanism against pathogens and herbivores, polyphenol describes a compound with a chemical structure containing one or more phenolic rings (Frank et al., 2020). Further classifications can be made based upon phenolic ring numbers, and associated structures binding these rings together (e.g., oxygenated heterocycle) (Cutrim and Cortez, 2018). With an estimated 15,000 varieties currently identified, flavonoids represent a considerably diverse class of polyphenol (Xiao, 2017). The structural basis of the flavonoid is a 15-carbon atom structure, forming a C6–C3–C6 heterocyclic skeleton, consisting of two benzene rings, linked to a heterocyclic ring (flavon ring). Ubiquitous in plant-based diets, flavonoids are the main polyphenolic component of citrus fruits. Differing heterocyclic ring oxidization gives rise to further subclasses: flavanones, flavonols, flavones, and anthocyanins (present in blood oranges). Citrus fruits (Rutaceae) such as oranges, grapefruit, lemons and limes are a particularly rich source of flavanones and are therefore sometimes called citroflavonoids. Flavanones are also present in other food sources such as seasoning herbs (e.g., rosemary, oregano, peppermint), but in much lower concentrations (Rothwell et al., 2013).

**FIGURE 1:**
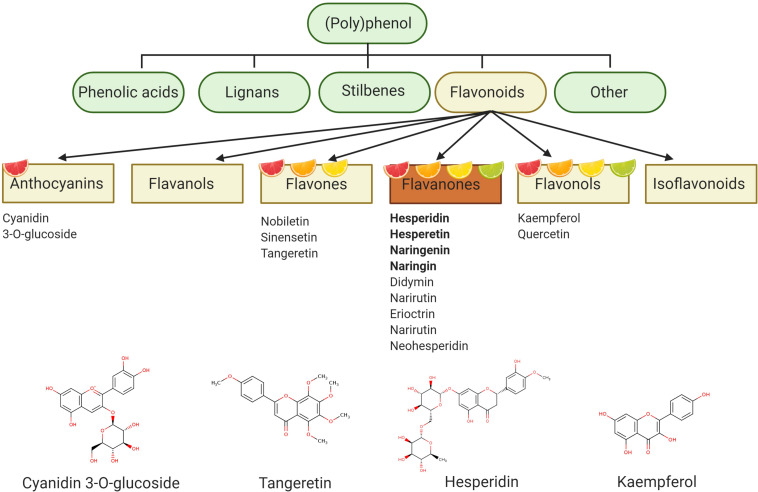
Polyphenol categorization – polyphenol describes a compound with a chemical structure containing one or more phenolic rings and can be classified into four main groups: phenolic acids, lignans, stilbenes, and flavonoids. Flavonoids represent the main polyphenolic component of citrus fruits and can be subdivided into six further subgroups. Flavanones are particularly abundant in citrus fruits and include hesperidin as one of the major flavanones. Figure created with BioRender.com.

Hesperidin (hesperetin-7-*O*-rutinoside) is a major citrus flavanone found in all citrus fruits (Jadeja and Devkar, 2014), reaching concentrations of 25.8–38.3 g/kg in sweet oranges (Iglesias-Carres et al., 2019) and up to 520 mg/L in orange juice (De Rycker et al., 2020). Hesperidin is also the most common of the flavonoid monomers in European diets with an intake of approximately 27 mg/daily (Zamora-Ros et al., 2015), although regional variations are observed (Wang et al., 2015a). Oranges and orange juices also contain naringenin-7-*O*-rutinoside (narirutin) in addition to smaller quantities of hesperetin-7-*O*-rutinoside-3′-*O*-glucoside, 4′-*O*-methyl-naringenin-7-*O*-rutinoside (didymin), and eriodictyol-7-*O*-rutinoside (eriocitrin) (Kay et al., 2017). Such compounds are believed to be responsible for many citrus related biological actions, although several other bioactives are also present in various citrus fruit and juice sources (e.g., anthocyanins, flavonols, carotenoids, pectins), and are therefore likely to also possess bioactive properties (Figure 1).

## Markers of Brain Health and Function

Markers of brain health and disease are key to understanding and treating neurological conditions. Development of sophisticated neuroimaging techniques [e.g., Positron Emission topography (PET) and functional Magnetic Resonance Imaging (fMRI)] has enabled subtle differences in brain activity, metabolism and structure to be monitored and distinguished between healthy and disease states (Agosta et al., 2013). Future incorporation of artificial intelligence techniques, e.g., deep learning, will likely enhance the prospect and feasibility of early detection of neurological conditions (Noor et al., 2020). However, the cost, expertise, and scalability of neuroimaging limits widespread use. Therefore, biological markers, detectable in the periphery (commonly cerebral spinal fluid or blood) remain equally important, and better suited for screening purposes. Below we discuss some specific markers of brain health in more detail.

### Neurotrophins

Neurotrophins are blood-brain barrier permeable signaling molecules with a crucial role in synaptic plasticity, neuronal cell survival and neurogenesis/synaptogenesis (Gibon and Barker, 2017). The presence of neurotrophins in the peripheral circulation makes them an ideal candidate for monitoring brain health and function. Of the neurotrophins, brain-derived neurotrophic factor (BDNF) has been the most extensively studied in the context of brain health, with peripheral levels associating with cognitive performance and neurological diseases (Nascimento et al., 2014; Küster et al., 2017; Palasz et al., 2020). BDNF levels have been reported to increase linearly to the age of 65, after which their levels markedly decrease (Neshatdoust et al., 2016). Interestingly, this predominantly corresponds to various MRI measures of brain health and cognition, although a high level of inter-study heterogeneity exists (McPhee et al., 2020).

### Blood-Brain Barrier

Loss of blood-brain barrier (BBB) integrity/function is considered an early process of neurodegenerative disease. Thus, markers of BBB permeability and/or breakdown are being actively explored as measures of brain health and disease risk. Leakiness of BBB can be determined using MRI (dynamic contrast-enhanced MRI) or PET with various tracers such as gadolinium or 18F-fluoro-2-deoxyglucose (FDG) respectively (Sweeney et al., 2018). Additionally, MRI (T2^∗^-weighted and susceptibility-weighted imaging MRI) can detect a chronic accumulation of small blood products, ‘microbleeds,’ thought to be linked to BBB breakdown. Peripheral markers of BBB function have also been established. CSF soluble platelet-derived growth factor receptor β (sPDGFRβ), shed from pericytes (which unsheathe the endothelium of brain capillaries) in response to injury, correlates with neurodegenerative disease progression and BBB breakdown (Miners et al., 2019; Nation et al., 2019; Montagne et al., 2020). Similarly, the ratio of CSF albumin to serum albumin, is frequently used as an indicator of BBB breakdown (Musaeus et al., 2020), although conflicting results have been reported (Skillbäck et al., 2017). Less invasive methods including measuring circulating tight junction proteins (occludin, ZO-1 and CLDN5) have also been forwarded as BBB breakdown measures (Jiao et al., 2015; Zhu et al., 2017), whilst serum 14-kDa monomeric form of transthyretin (TTR) and serum concentrations of the astrocytic protein S100B, have been suggested as measures of BBB damage (Marchi et al., 2004).

### Metabolic Function

Metabolic homeostasis is particularly important for brain health and function due to the energy-demanding nature of the brain. Brain energy metabolism declines subtly during the aging process and prior to neurological disease diagnosis (Zilberter and Zilberter, 2017), and accumulating evidence demonstrates how this impairment of energy metabolism can exacerbate neurodegenerative disease progression (Cunnane et al., 2016; Camandola and Mattson, 2017). Neuroimaging techniques can effectively indicate metabolic disturbances. For example, FDG-PET is frequently utilized to determine brain glucose metabolism rates across the brain, which subsequently correlate to synaptic activity and disease risk (Shivamurthy et al., 2015). A multitude of other positron-emitting isotope tracers exist, including [1-^11^C]-DHA (Yassine et al., 2017), and the incorporation of such tracers can hone in on specific aspects of brain metabolism which may be disease-specific. Furthermore, the ratio of *N*-acetylaspartate (NAA) and myo-inositol (MI), two abundant metabolites in the human brain, has been reported to be a good predictor of mild cognitive impairment in cognitively normal older adults as measured by ^1^H MRS in the posterior cingulate cortex (PCC) (Kantarci et al., 2013). Finally, peripheral measures of metabolic function such as insulin resistance and blood glucose, are routinely determined in clinical trials in which cognitive health is a primary output, and have been found to correlate with cognitive decline (Wium-Andersen et al., 2019), and brain atrophy (Erus et al., 2015).

### Inflammatory Markers

Although neuroinflammation is an essential part of the brain response to infection or injury (Glass et al., 2010), sustained neuroinflammatory processes may contribute to the progressive neuronal damage observed in aging (Barrientos et al., 2015) and aged-related cognitive disorders (Heppner et al., 2015; Herrero et al., 2015). As resident macrophages, activated microglial cells have the capacity to synthesize a wide range of pro-inflammatory and anti-inflammatory cytokines and molecular mediators which contribute to the systemic inflammatory milieu and to the progression of neurodegenerative disease (Perry and Holmes, 2014). As an integral component of numerous neurological diseases, effective monitoring of neuroinflammation would be highly advantageous. PET may represent a viable option to achieve this (Chandra et al., 2019; Kreisl et al., 2020), along with a range of markers for activated microglia such as Translocator protein (TSPO) radioligands showing promise both in a preclinical setting and in humans. Optimization of TSPO tracers and identification of more specific neuroinflammatory tracers is, however, warranted to improve upon accuracy (Kreisl et al., 2020). Similar to peripheral markers of metabolic function, although not a direct marker of brain inflammation, circulating levels of the pro-inflammatory (IL-1α, IL-1β, IL-6, TNF-α) and anti-inflammatory cytokines (IL-1ra and IL-10) indicate a chronic inflammatory environment and probably contribute to brain inflammation. Again, these measures frequently correlate with measures of brain health and disease such as cognitive performance (Magalhães et al., 2018; Contreras et al., 2020; Ribeiro-Santos et al., 2020).

### Cognitive Testing and Sensory Parameters

As well as biological and neuroimaging techniques, brain health can be determined through cognitive assessment. For this, a wide range of tests can be employed, usually in combination as a battery, to address multiple aspects of cognition such as memory, language, executive functions and attention. Memory, a common target of numerous neurodegenerative disorders, can be separated into specific memory domains, enabling researchers to pinpoint disruptions to specific brain systems (e.g., episodic memory; hippocampal–diencephalic system, working memory; dorsolateral prefrontal cortex, semantic memory; anterior temporal lobe, executive functioning; frontal lobe and beyond) (Kipps and Hodges, 2005). Traditionally, such examination has required extensive clinical testing, performed by trained individuals (Kipps and Hodges, 2005), however, recent advancements in technology, e.g., smartphones and internet-based cognitive assessment tools may enable screening to take place outside of a clinical setting (Coutrot et al., 2019; Hays et al., 2019; Sternin et al., 2019).

Finally, it is worth briefly mentioning sensory parameters, particularly olfaction, which are potentially underrated, and certainly overlooked markers of brain health, despite consistent reports of their predictive capacity in cognitive decline (Brai et al., 2020).

## Bioavailability of Citrus Flavonoids

The extent to which citrus flavanones may exert their biological action is strongly related to their bioavailability which can be affected by many factors including the structure of the compound, the food matrix, or host factors (age, sex, genetic polymorphism, gut microbiota etc.) (Kay et al., 2017; Morand and Tomás-Barberán, 2019). There is an apparent difference between the absorption of glycosides and aglycones with the glycosylation of flavanones increasing their hydrophilicity therefore abolishing passive diffusion and thus lowering their bioavailability (Najmanová et al., 2019). For example, following oral administration of hesperidin, plasma concentrations of flavanone conjugates (e.g., hesperetin-glucuronides and sulfo-glucuronides) were detected after 3 h and reached a peak between 5 h and 7 h (Manach et al., 2003), highlighting that the main site for flavanone absorption is the small intestine and the colon. Such results were further confirmed by a more recent study where fasted participants, aged 51–69 years, received either orange juice or a hesperidin supplement (both providing 320 mg hesperidin) versus a control (all matched for sugars and vitamin C content). Total plasma flavanone metabolite concentrations were significantly higher 5 h after the orange juice intervention than after control with hesperidin-glucuronide and naringenin-7-*O*-glucuronide, largely contributing to the total plasma flavanone concentration. Unexpectedly, no significant concentration of hesperidin metabolites was observed at 5 h following the hesperidin supplement intake, which may highlight the importance of food matrix in the bioavailability of these compounds (Schär et al., 2015). In addition to the main phase II metabolites found in the systemic circulation, the intestinal microflora further degrades aglycones into smaller phenolics. For example, the main degradation product of hesperetin is 3-(3′-hydroxy-4′-methoxyphenyl)propionic acid, although many other compounds have also been reported (see Kay et al., 2017; Najmanová et al., 2019 for extensive reviews of flavanones metabolism).

Flavanone bioavailability can differ across citrus source and/or form. For example, the bioavailability of hesperidin, indirectly determined from excreted hesperetin (main hesperidin metabolite marker) is comparable for both whole orange fruit (1.5%) and orange juice (2.9%), despite the considerably higher flavanone content of the fruit (Aschoff et al., 2016), reflecting potential absorption and metabolic differences. Fresh and packaged orange juice, on the other hand, appear to have relatively similar metabolic kinetics in regards to flavanones, and therefore the higher flavanone content found in packaged juice (machine pressed) translates to a threefold greater flavanone status (Silveira et al., 2014).

In addition, a large inter-individual variability in the bioavailability of citrus flavanones has been reported with high, medium, and low flavanone metabolite excreters identified following intake of citrus flavanones and citrus juices (Nielsen et al., 2006; Brett et al., 2008; Vallejo et al., 2010; Aschoff et al., 2016). One of the key factors in this variability may reside in the host–microbiota diversity necessary for the conversion of the flavanone rutinoside into their aglycone forms (Stevens et al., 2019), although the impact of lifestyle factors such as exercise have also been reported (Pereira-Caro et al., 2017). Despite the demonstrated high inter-individual variability in citrus flavanones metabolism, limited evidence is currently available regarding the role of the gut microbiota composition and no correlation with the effects on brain health biomarkers has been demonstrated.

When considering the bioavailability of citrus flavonoids from a neurological perspective, one must also consider the compounds ability to traverse the BBB. The extent to which specific citrus polyphenols cross the BBB remains to be fully categorized, however, evidence to date in the form of *in vitro* cellular models (e.g., RBE4, b.END5, and hCMEC/D3) (Youdim et al., 2003; Yang W. et al., 2014), and preclinical approaches (Peng et al., 1998; Tsai and Chen, 2000; Youdim et al., 2004) indicate that citrus flavonoids, namely; hesperetin, naringenin, as well as their relevant metabolites, are able to reach the brain. Whether these models fully translate to humans, particularly when ingested with other complex food sources is yet to be proven, however, pursuing this question will undoubtedly enhance our understanding of citrus polyphenols in the context of brain health and disease, elucidating the extent to which their bioactivity stems from direct interaction with the brain parenchyma.

## Microbiota: Gut: Brain Axis

A healthy gastrointestinal tract is a nutrient-rich environment hosting approximately 100 trillion microbes (Lin and Zhang, 2017), that constitute the gut microbiota. Gut microbial composition is increasingly recognized as a central factor in health and disease, protecting the intestinal gut barrier and preventing the establishment of pathogenic microorganisms. The gut microbiota also provides a large repertoire of genes, antigens and metabolites that can regulate immune and metabolic functions. The gut-brain axis describes a bidirectional system that encompasses both neuro-immune (Teratani et al., 2020) and neuro-endocrine communication as well as a direct neuronal connection (vagus nerve) (Tan et al., 2020), with each mode of transmission receiving microbial modulation (Lv et al., 2019; Wang et al., 2019). The gut-brain axis has rapidly emerged in recent decades, with an influx of literature implicating gut health and microbial dysbiosis with specific neurological diseases/conditions, although the multi-factorial nature and complexity of these diseases/conditions has left mechanistic validation somewhat lagging (Sherwin et al., 2018). Indeed, further elucidation of the mechanistic as well as the determination of overall therapeutic validity are still required (Peterson, 2020). An area in which mechanistic evidence is beginning to gain traction is the synthesis and/or regulation of metabolites by the gut-microbiota, with many derivative metabolites established as neurochemicals or possessing neuromodulatory properties. Short-chain fatty acids (SCFA) are a relatively well-characterized example of this (Dalile et al., 2019) and are produced via fermentation of complex carbohydrates (dietary fibers). SCFA levels are diminished in response to antibiotic treatment (Høverstad et al., 1986) and similarly low levels tend to associate with conditions such as depression, Alzheimer’s (AD) and Parkinson’s disease (Dalile et al., 2019). For example, the SCFA propionate was recently reported to inhibit pathways associated with non-specific microbial infections via a CD14-dependent mechanism, to suppress the expression of LRP-1 and to protect the BBB from oxidative stress via NRF2 (*NFE2L2*) signaling (Hoyles et al., 2018). The influence of metabolites such as SCFA produced by our ‘gut-biofactory’ has been extensively reviewed by Skonieczna-Żydecka et al. (2020).

The considerable inter-individual variability of the gut microbiota combined with its apparent modifiable/dynamic nature has led to it being identified as a therapeutic target. The fact that beneficial shifts could be achieved through non-invasive, relatively safe interventions, e.g., prebiotics, make it an attractive target. The gut microbiota is both modulated by, and modulates, polyphenolic compounds (Koudoufio et al., 2020). Citrus polyphenols appear to be no exception, with neohesperidin recently shown to reverse high-fat-diet-induced intestinal microbiota dysbiosis by increasing general microbial diversity as well as specific strains including *Bacteroidetes* and *Firmicutes* (Lu et al., 2020). A similar experiment in which hesperidin was administered exerted similar prebiotic effects with treated mice displaying improved metabolic maker profile (Guirro et al., 2020). These beneficial hesperidin mediated effects are in agreement with an earlier experiment, which interestingly reported upon the concomitant immunomodulatory actions (Estruel-Amades et al., 2019). In healthy volunteers, continuous consumption of commercial pasteurized orange juice for 2 months improved blood biochemical parameters, such as low-density lipoprotein-cholesterol, glucose, and insulin sensitivity and positively modulated the composition and metabolic activity of the microbiota, increasing the population of fecal *Bifidobacterium* spp. and *Lactobacillus* spp. (Lima et al., 2019). In addition, daily consumption of 500 mL of two Brazilian orange juices (e.g., Cara Cara and Bahia juices) for 7 days increased the abundance of *Mogibacteriaceae*, *Tissierellaceae*, *Veillonellaceae*, *Odoribacteraceae*, and *Ruminococcaceae* families in healthy volunteers (Brasili et al., 2019). Furthermore, daily consumption of 300 ml of orange juice for 60 days affected the levels of *Lactobacillus* spp., *Akkermansia* spp., and *Ruminococcus* spp. and improved the glycemia and lipid profiles in 20–35 years old healthy female volunteers (Fidélix et al., 2020). These recent reports emphasize the prebiotic potential of citrus polyphenols, particularly in metabolic disease, with no specific information related to brain functions (e.g., gut-brain axis). With this being said, it must be mentioned that current evidence has been predominantly provided through preclinical and in particular, rodent experiments. Therefore, current perspectives derived from these studies should be cautiously interpreted until validated through robust clinical trials since fundamental human to mouse differences, such as microbial composition and gut physiology, may render direct translation inappropriate.

## Method

A non-systematic search was performed in PubMed using the following keywords to retrieve preclinical and clinical data: [(Dementia OR depression OR Alzheimer OR Parkinson OR neurodegeneration OR neuroinflammation) AND (citrus polyphenol OR flavanone OR hesper^∗^ OR naring^∗^) AND (cognition OR brain)]. Articles in English or with English abstracts were retrieved. All evidence was read and chosen based on the Authors’ evaluation of relevance. The search was concluded in August 2020 and updated in November 2020, and included all articles present in PubMed, without temporal limits.

## Preclinical Evidence for Citrus Flavonoids and Brain Health/Function

Neurodegeneration describes a process of progressive cell dysfunction and eventual neuronal cell death. In diseases such as late-onset Alzheimer’s Disease (LOAD) this process can span decades. Although usually categorized based upon distinct pathologies, the overall process of neurodegeneration is one that is multi-factorial, with many neurodegenerative diseases sharing common pathogenic mechanisms underpinning disease progression (Jellinger, 2010), potentially explaining the lack of efficacy of single target drugs. These common mechanisms associated with neurodegeneration include: neuroinflammatory/neuroimmune responses, oxidative stress, impaired bioenergetics/mitochondrial dysfunctions, dysfunction of neurotrophins and abnormal protein dynamics. The mechanistic understanding of citrus polyphenols in brain health stems largely from preclinical and *in vitro* studies. The focus of these preclinical studies often centers on mitigating specific disease pathologies. Indeed, a wealth of preclinical evidence has highlighted the multifaceted nature of citrus polyphenols neurologically. Interestingly, as highlighted in Table 1, the major citrus polyphenols share common mechanistic actions, overlapping considerably with the aforementioned deficits associated with neurodegeneration. Below we discuss these mechanisms in more details focusing on the key citrus flavonoids and in particular on the flavanones hesperidin and naringin along with their aglycone forms (i.e., hesperetin and naringenin) and the flavonol, kaempferol.

**TABLE 1:** Overview of the molecular mechanisms underlying the impact of citrus flavonoids on brain health and disease in preclinical models.

Citrus Polyphenol	Mechanism	Proposed mode of action	Reported effective Dose (mg/Kg body weight)	Model
**Hesperidin**	Anti-inflammatory	**↓**NF-κB, **↓**TNF-α, **↓** IL-1β	100, 200 (15 days) Dose dependant with 200 more effective	Sodium arsenite treatment, 10-week-old male Sprague-Dawley rat (Kuzu et al., 2020)
		**↓**TNF-α, **↓**IFNγ, **↓**IL-1β, **↓**IL-2, **↓**IL-6, ↑IL-10	50 (28 days)	Intrastriatal injection 6-OHD, 3–6-month adult male C57BL/6 mouse (Antunes et al., 2020)
		↓TNF-α, ↓IL-1β, ↓IL-6, ↓HMGB1, ↓RAGE, ↓p-NFκB, ↓p-IκBa	100 and 200 (three times a week for 3 weeks)	Chronic unpredictable mild stress, 6–8-week ICR male mouse (Fu et al., 2019)
		↓IL-1β, ↓TNF-α	50(14 days)	Concussive head injury, 10–11-week male NMRI mouse (Kosari-Nasab et al., 2018)
		↓IL-1β, **↓**IL-6	50 (14 days)	Olfactory bulbectomy, male C57BL/6 mouse (Antunes et al., 2016)
		↓IL-1β, ↓IL-6, ↓TNF-α, ↑miRNA132	25, 50, 100 (7 days)	I.P. LPS, male ICR mouse (Li M. et al., 2016)
		↓Iba-1, ↓TGF-β	100 (10 days)	Transgenic APP/PS1, 5-month male mouse (Li et al., 2015)
		**↓**GFAP, **↓**iNOS, **↓**NF-κB, **↓**COX-2 (No quantification)	100 and 200 (15 days)	Swiss male albino mouse (Javed et al., 2015)
		↓IL-6	25, 50, and 100 (21 days)	I.P. STZ, male albino Wistar rat (El-Marasy et al., 2014)
		↓IL-1β, ↓TNF-α, **↓**GFAP, **↓**iNOS	50 (15 days)	Middle cerebral artery occluded, 16-week male Wistar rat (Raza et al., 2011)
	Anti-oxidative	↓MDA, ↓8-OHdG, ↑GSH, ↑GPx, ↑SOD, ↑CAT	100, 200 (15 days) Dose dependant with 200 more effective	Sodium arsenite treatment, 10-week-old male Sprague-Dawley rat (Kuzu et al., 2020)
		↑Glo1, ↓AGE, ↓ROS, ↓MDA, ↑GSH, ↑SOD, ↑Nrf2, ↑γ-GCS	50, 150 (10 weeks) Dose dependant with 150 more effective	I.P. STZ, adult male Sprague Dawley rat (Zhu et al., 2020)
		↑Nrf2, ↓MDA, ↑SOD activity, ↑GPx activity, ↑CAT activity	100 (21 days)	IV injection Methotrexate, 4–5 weeks male Sprague Dawley rat (Welbat et al., 2020)
		↓MDA, ↓NO, ↑GSH, ↑GSH-Px, ↑SOD, ↑CAT	10 (21 days)	Acrylamide treated, adult male Wistar albino rat (Elhelaly et al., 2019)
		↓MDA	50↑(14 days)	Concussive head injury, 10–11-week male NMRI mouse (Kosari-Nasab et al., 2018)
		↑MDA, ↓GSH, ↓SOD, ↓CAT	25, 50, 100 (14 days) Dose dependant with 100 more effective	L-Methionine treated, male Wistar rat (Hemanth Kumar et al., 2017)
		↓TBARS, ↑GSH, ↑SOD, ↑CAT, ↑GPx	100 (60 days)	I.P. AlCl3, 10–12-week male albino Wistar rat (Justin Thenmozhi et al., 2017)
		↓TBARS, ↑GSH	100 and 200 (15 days) Dose dependant with 200 more effective	Intracerebroventricular injection STZ, 12-month Swiss male albino mouse (Javed et al., 2015)
		↓MDA, (↑GSH did not increase in 100 group)	25, 50, and 100 (21 days)	I.P. STZ, male albino Wistar rat (El-Marasy et al., 2014)
		↓H2O2, ↓MDA, ↑GSH, ↑T-AOC	100 (16 weeks)	APPswe/PS1dE9, 3-month-old male mouse (Wang et al., 2014)
		↓ROS, ↑GPx, ↑GSH, ↑TRAP, ↑CAT, ↑GPx, ↓GR (SOD and GST no change)	50 (28 days)	Intracerebroventricular injection of 6-OHDA, female C57BL/6 mouse (Antunes et al., 2014)
		↓TBARS, ↑NP-SH, ↑GSH, ↑GST, ↑GR, ↓XO	50 (28 days)	I.P. STZ, male Wistar rat (Ashafaq et al., 2014)
		↓TBARS, ↑GPx, ↑SOD, ↑CAT	50 (15 days)	Middle cerebral artery occluded, 16-week male Wistar rat (Raza et al., 2011)
	Anti-Apoptotic/Proliferative	↓Caspase-3	100, 200 (15 days) Dose dependant with 200 more effective	Sodium arsenite treatment, 10-week-old male Sprague-Dawley rat (Kuzu et al., 2020)
		↑BDNF, ↓p21 positive cells, ↑DCX	100 (21 days)	I.V injection Methotrexate, male Sprague Dawley rat (Welbat et al., 2020)
		↑BDNF, ↑NGF, ↑NT3	50 (28 days)	Intrastriatal injection 6-OHD, 3–6-month adult male C57BL/6 mouse (Antunes et al., 2020)
		↑Ki-67, ↑BrdU, ↑DCX	100 (21 days)	IV injection Methotrexate, male Sprague Dawley rat (Naewla et al., 2019)
		↑BDNF, ↑p-TrkB	100, 200 (3x in 21 days)	Chronic unpredictable mild stress, 6–8-week male ICR mouse (Fu et al., 2019)
		↑BDNF	100 and 200 (21 days)	Concussive head injury, 10–11-week male NMRI mouse (Kosari-Nasab et al., 2018)
		↑BDNF, ↑NGF	50 (14 days)	Olfactory bulbectomy, male C57BL/6 mouse (Antunes et al., 2016)
		↓BAX, ↑BCL-2	100 (60 days)	IP AlCl3, 10–12-week male albino Wistar rat (Justin Thenmozhi et al., 2017)
		↑p-ERK, ↑BDNF (ERK dependent)	25, 50 (3 weeks)	Chronic mild stress, 5-week male ICR mouse (Li C. F. et al., 2016)
		↑BDNF	25, 50, and 100 (21 days)	I.P. STZ, male albino Wistar rat (El-Marasy et al., 2014)
	Bioenergetic	↓AChE	100, 200 (15 days) Dose dependant with 200 more effective	Sodium arsenite treatment, 10-week-old male Sprague-Dawley rat (Kuzu et al., 2020)
		↓AChE	50 (14 days)	Olfactory bulbectomy, male C57BL/6 mouse (Antunes et al., 2016)
		**↓**AChE activity	100 and 200 (15 days)	Intracerebroventricular injection STZ, 12-month Swiss male albino mouse (Javed et al., 2015)
		↑ MT complex (I-IV) activity, ↑MTT activity, ↑GSK3b phos	100 (16 weeks)	APPswe/PS1dE9, 3-month-old male mouse (Wang et al., 2014)
		↓AChE, ↑Na + -K + ATPase	50 (28 days)	I.P. STZ, male Wistar rat (Ashafaq et al., 2014)
	Proteinopathy	↓Aβ deposition	100 (10 days)	Transgenic APP/PS1–21, 5-month male mouse (Li et al., 2015)
		No change Aβ deposition	100 (16 weeks)	Three-month-old male, APPswe/PS1dE9 mouse (Wang et al., 2014)
	BBB dysfunction	↓EB leakage, ↓claudin-5 and ZO-1 disturbance	10 (1 dose prior to MCAO)	Left middle cerebral artery occlusion, male 8 weeks ICR mouse (Lee et al., 2020)
**Hesperetin**	Anti-inflammatory	↓TLR4, ↓GFAP, ↓Iba-1, ↓TNF-α, ↓IL-1β, ↓p-NF-κB	50 (5 weeks)	I.P. LPS, 7/8 weeks male C57BL/6N mouse (Muhammad et al., 2019)
		↓TNF-a, ↓IL-1b, ↓iNOS	20 (8 days)	Hippocampal injection Kainic Acid, mouse (Kwon et al., 2018)
		↓GFAP, ↓NF-κB	50 (1 week)	Intrastriatal injection 6-OHDA, male adult Wistar rat (Kiasalari et al., 2016)
	Anti-oxidative	↓MDA, ↑GSH, ↑SOD, ↑CAT (50 only), ↓NO (50 only)	5, 50 (3 days)	I.P. scopolamine, male albino mouse (Ishola et al., 2019)
		↑Nrf-2, ↑HO-1 ↓ROS, ↓LPO	50 (5 weeks)	I.P. LPS, 7–8-week male C57BL/6N mouse (Muhammad et al., 2019)
		↓MDA, (GSH, CAT, SOD, GPx, GRx No change) (however effect significant in Nano form)	10, 20 (3 weeks)	I.P. injection STZ, male Wistar rat (Kheradmand et al., 2018)
		↓TBARS, ↓LOOH and ↓Protein carbonyl content (LPO), ↓ OH-, ↓NO, ↑GSH, ↑TSH, ↑SOD, ↑CAT, ↑GPx and ↑GST	40 (21 days)	Subcutaneous injection of Cadmium, male albino Wistar rat (Shagirtha et al., 2017)
		↓MDA, ↑CAT, ↑GSH, (NO No change)	50 (1 week)	Intrastriatal injection of 6-OHDA, male adult Wistar rat (Kiasalari et al., 2016)
	Anti-Apoptotic/Proliferative	↑BDNF	5, 50 (3 days)	I.P. scopolamine, male albino mouse (Ishola et al., 2019)
		↓p-JNK, ↓Bax:Bcl2, ↓Caspase-3	50 (5 weeks)	I.P. LPS 7-8-week, male C57BL/6N mouse (Muhammad et al., 2019)
		↓Bax, ↓cytochrome c, ↓caspase 3 and 9, ↑Bcl2	40 (21 days)	Subcutaneous injection of Cadmium, male albino Wistar rat (Shagirtha et al., 2017)
		↑Bcl2, ↓DNA fragmentation	50 (1 week)	Intrastriatal injection of 6-OHDA, male adult Wistar rat (Kiasalari et al., 2016)
	Bioenergetic	↓AChE	5, 50 (3 days)	I.P. scopolamine, male albino mouse (Ishola et al., 2019)
		↑AChE, ↑ATPases, ↑ MT complex (I-IV) activity	40 (21 days)	Subcutaneous injection of Cadmium, male albino Wistar rat (Shagirtha et al., 2017)
	Proteinopathy	↑Cathepsin, trypsin and pronase activity	40 (21 days)	Subcutaneous injection of Cadmium, male albino Wistar rat (Shagirtha et al., 2017)
**Naringenin**	Anti-inflammatory	↓GFAP, ↓TNF−α, ↓IL−1β, ↑IL10	250–300 (10 weeks)	High-fat diet-fed, 10-month SAMP8 male mouse (Zhou et al., 2020)
		↓iNOS	25, 50, 100 (5 days)	I.P. MPTP, male C57BL/6J mouse (Sugumar et al., 2019)
		↓IL−6, ↓TNF−α, ↓NFκB, ↓IFN-γ	50, 100 (14 days)	Bilateral olfactory bulbectomy, 3-month BALB/c male mouse (Bansal et al., 2018)
		↓NFκB, ↓TNFα, ↓COX2, ↓iNOS, ↓TLR4, ↓GFAP	100 (7 days)	I.P. LPS, male albino Wistar rat (Khajevand-Khazaei et al., 2018)
		↓IL−1β, ↓TNF−α (not 25 mg/kg)	25, 50, 100 (5 days)	I.P. MPTP, male C57BL/6J mouse (Mani et al., 2018)
		↓NFκB, ↓iNOS, ↓COX2, ↓TNF−α, ↓IL−1β	50 (21 days)	Middle cerebral artery occlusion, 16-week male Wistar rat (Raza et al., 2013)
	Anti-oxidative	↑SOD, ↑GSH, ↓MDA, ↓NO	250–300 (10 weeks)	High-fat diet-fed, 10 months SAMP8 male mouse (Zhou et al., 2020)
		↓SOD activity, ↑CAT, ↑GPx,↑GSH, ↓MDA	50 (14 days)	I.P. AlCl_3_ + D-gal injection, young albino Wistar rat (Haider et al., 2020)
		↑GSH, ↑GST, ↓MDA, ↓Protein carbonyl content	100 (15 days)	Oral MeHg, 5-week male Swiss albino mouse (Krishna Chandran et al., 2019)
		↓LPO, ↑CAT, ↑GR	25, 50, 100 (5 days)	I.P. MPTP, male C57BL/6J mouse (Sugumar et al., 2019)
		↑SOD, ↑GSH, ↑CAT, ↓MDA, ↓NO	50, 100 (14 days)	Bilateral olfactory bulbectomy, 3-month BALB/c male mouse (Bansal et al., 2018)
		↑Nrf2, ↑CAT, ↑SOD, ↑GSH, ↓MDA	100 (7 days)	I.P. LPS, male albino Wistar rat (Khajevand-Khazaei et al., 2018)
		↑SOD, (↑GSH not significant), (↓NO 100 mg/kg not significant)	25,50,100 (5 days)	I.P. injection MPTP, male C57BL/6J mouse (Mani et al., 2018)
		↑CAT, ↑SOD, ↑GSH, ↓MDA, ↑GPx, ↓MDA	50 (16 days)	Young adult male Albino Wistar rat (Liaquat et al., 2018)
		↓MDA, (SOD and NO No change)	100 (single dose)	Dorsal hippocampal Aβ injection, adult male Wistar rat (Ghofrani et al., 2015)
		↓NOD2, ↓RIP2, ↓MMP-9	100 (4 days)	Permanent middle cerebral artery occlusion, male Sprague–Dawley rats (Bai et al., 2014)
		↓ROS, ↓NO, ↓MDA, ↓PCO, ↑ascorbic acid, ↑total thiol groups ↓SOD, ↓CAT, (GPx No change)	50 (4 weeks)	I.P. iron (Fe) injection, 10-week male Wistar rat (Chtourou et al., 2014)
		↑Nrf2 and downstream ARE genes, ↓ROS, ↑GSH	70 (4 days)	Intastriatal 6-OHDA, 10-week male C57BL/6 mouse (Lou et al., 2014)
		↓TBARS, ↑GSH, ↓NO, ↓MPO activity, ↑SOD activity	50 (21 days)	Middle cerebral artery occlusion, 16-week male Wistar rat (Raza et al., 2013)
	Anti-Apoptotic/Proliferative	↑BDNF, ↑Shh	50 (5 weeks)	Chronic unpredictable mild stress, adult male Wistar rat (Tayyab et al., 2019)
		↑BDNF	50, 100 (14 days)	Bilateral olfactory bulbectomy, 3-month BALB/c male mouse (Bansal et al., 2018)
		↓DNA Fragmentation	100 (single dose)	Dorsal hippocampal Aβ injection, adult male Wistar rat (Ghofrani et al., 2015)
		↓p-JNK, ↓p-p38	70 (4 days)	Intastriatal 6-OHDA, 10-week male C57BL/6 mouse (Lou et al., 2014)
		↑BDNF (Hippocampus)	10, 20 (3 Weeks)	Chronic unpredictable mild stress, 5-week male ICR mouse (Yi et al., 2014)
	Bioenergetic	↑Ach, ↓AChE, ↓AChE activity	50 (14 days	I.P. AlCl_3_ + D-gal injection, young albino Wistar rat (Haider et al., 2020)
		↑ MT complex (I-IV) activity, ↓MT DNA damage	100 (15 days)	Oral MeHg, 5-week male Swiss albino mouse (Krishna Chandran et al., 2019)
		↓KYN:TRP ratio	50, 100 (14 days)	Bilateral olfactory bulbectomy, 3-month BALB/c male mouse (Bansal et al., 2018)
		↓AChE activity	100 (7 days)	I.P. LPS, male albino Wistar rat (Khajevand-Khazaei et al., 2018)
		↑Ach, ↓AChE	50 (16 days)	Young adult male albino Wistar rat (Liaquat et al., 2018)
		↑Na + -K + ATPase, ↑AChE activity	50 (4 weeks)	I.P. injection iron (Fe), 10 week male Wistar rat (Chtourou et al., 2014)
		↑PPARγ↑IDE, ↑INS, ↑INSR	25, 50, 100 (21 days)	Intracerebroventricular injection STZ, adult male Sprague Dawley rat (Yang W. et al., 2014)
	Proteinopathy	↓Soluble and insoluble Aβ 40, Aβ 42, ↓APP, ↓BACE1, ↓P−tau, ↓P−GSK	250–300 (10 weeks)	High-fat diet-fed, 10-month SAMP8 male mouse (Zhou et al., 2020)
		↓αSYN (100 mg/kg only), ↓αSYN + cells	25, 50, 100 (5 days)	I.P. injection MPTP, male C57BL/6J mouse (Mani et al., 2018)
		↓GSK-3β, ↓pTau/Tau ratio, ↓Aβ42	25, 50, 100 (21 days)	Intracerebroventricular injection STZ, adult male Sprague Dawley rat (Yang W. et al., 2014)
**Naringin**	Anti-inflammatory	↓GFAP	100 (14 days)	STZ treated, C57BL/6, 9-week male mouse (Okuyama et al., 2018)
		↓TNF-α, ↓IL-1β, ↓IL-6, ↓NF-kB	40 and 80↑(28 days)	Intrastriatal injection of quinolinic acid, adult male Sprague-Dawley rat (Cui et al., 2018)
		↓TNF-α	20 and 40 (28 days)	Intastriatal injection collagenase, adult female Wistar rat (Singh et al., 2017)
		↓NF-kB, ↓GFAP	80 (14 days)	I.P. injection 3-nitropropionic acid, male Wistar rat (Gopinath and Sudhandiran, 2016)
		↓iNOS, ↓TNF-α, ↓NF-κB	25, 50 100 (5 weeks)	Cisplatin exposed, middle aged Wistar rat (Chtourou et al., 2015)
		↓TNF-α, ↓IL-1β	50, 100, and 200 (21 days)	Intracerebroventricular STZ, adult male Wistar rat (Sachdeva et al., 2014)
	Anti-oxidative	↑SOD, ↑GSH, ↓MDA, ↓NO	40 and 80 (28 days)	Intrastriatal injection quinolinic acid, adult male Sprague-Dawley rat (Cui et al., 2018)
		↓MDA (reduced by 10 mg/kg also), ↑CAT, ↑GSH, ↑SOD (40 mg/kg only), ↓NO	20 and 40 (28 days)	Intastriatal injection collagenase, adult female Wistar rat (Singh et al., 2017)
		↓MDA, ↓PCO, ↓H2O2, ↓ROS, ↓NO (50 and 100 only), ↑SOD, ↑CAT, ↑GSH, ↑GPx	25, 50, and 100 (5 weeks)	Cisplatin exposed, middle aged Wistar rat (Chtourou et al., 2015)
		↓ROS	100 (20 weeks)	High-fat diet induced cognitive decline, male C57BL/6 mouse (Wang et al., 2015b)
		↓MDA, ↓NO, ↑GSH, ↑SOD, ↑CAT	50, 100, and 200 (21 days)	Intracerebroventricular STZ, adult male Wistar rat (Sachdeva et al., 2014)
	Anti-Apoptotic/Proliferative	↑DCX	100 (14 days)	STZ treated, C57BL/6 male (9-week) mouse (Okuyama et al., 2018)
		↓Bax, ↑Bcl-2, ↓Caspase-3, ↑PPAR-γ	40 and 80 (28 days)	Intrastriatal injection of quinolinic acid, adult male Sprague-Dawley rat (Cui et al., 2018)
	Bioenergetic	↑MT complex (I-IV)	40 and 80 (28 days)	Intrastriatal injection of quinolinic acid, adult male Sprague-Dawley rat (Cui et al., 2018)
		↓AChE activity, ↑Na + -K + ATPase, ↑Ca2 + ATPase, ↑Mg2 + ATPase (increased by 25 mg/kg also)	50 and 100 (5 weeks)	Cisplatin exposed, middle aged Wistar rat (Chtourou et al., 2015)
		↑MT membrane potential, ↑ATP levels, ↑AMPK	100 (20 weeks)	High-fat diet induced cognitive decline, male C57BL/6 mouse (Wang et al., 2015b)
		↓AChE activity, ↑MT complex (I-IV) activity	50, 100, and 200 (21 days)	Intracerebroventricular STZ, adult male Wistar rat (Sachdeva et al., 2014)
	Proteinopathy	**↓**Thr231 and Ser396 Tau phos	100 (14 days)	STZ treated, C57BL/6 male (9-week) mouse (Okuyama et al., 2018)
**Kaempferol**	Anti-inflammatory	↓Iba-1, ↓TNFα, ↓IL-1β, ↓IL-5, ↓IL-6, ↓iNOS, ↓COX-2, ↓NF-κB	25, 50, 100 (7 days) Dose dependant 100 most effective	Cerebral ischemia/reperfusion, rat (Li et al., 2019)
		↓Iba-1, ↓IL-1β, ↓IL-6, ↓TNFα, ↓MCP-1, ↓ICAM-1, ↓COX-2, ↓HMGB1, ↓TLR4	20 and 50 (7 days) Dose dependant 50 most effective	I.P. LPS, adult male BALB/c mouse (Yang et al., 2019)
		↓Iba1, ↓IL-1β, ↓IL-6, ↓TNF-α, ↓MCP-1, ↓COX-2, ↓iNOS, ↓HMGB1, ↓TLR4, ↓MyD88	25, 50, or 100 (7 days)	I.P. LPS, adult male BALB/c mouse (Cheng et al., 2018)
		↓TNF-α	10 (21 days)	Intracerebroventricular injection STZ, OVX female Wistar rat (Kouhestani et al., 2018)
	Anti-oxidative	↑SOD, ↑GSH, ↓MDA	10 (21 days)	Intracerebroventricular injection STZ, OVX female Wistar rat (Kouhestani et al., 2018)
		↓MDA, ↑GPx, ↑CAT, ↑SOD, ↑GSK3β – Nrf2	21 (14 days)	I.P. Chlorpyrifos daily for 14 days, 8-week male albino Wistar rat (Hussein et al., 2018)
	Bioenergetic	↑AChE activity	21 (14 days)	I.P. Chlorpyrifos daily for 14 days, 8-week male albino Wistar rat (Hussein et al., 2018)
		↑TCA cycle flux, ↓acyl carnitines, ↑N-acetyl aspartate	1 (three times: 1, 24, 48 h)	Traumatic Brain Injury, 23–24-day male Wistar rat (Chitturi et al., 2019)
	BBB protection	↓EB leakage, ↓MMP-3	25, 50, 100 (7 days) Dose dependant 100 most effective	Cerebral ischemia/reperfusion, rat (Li et al., 2019)
		↓ultrastructure destruction (electron microscope), ↑occludin, ↑claudin-1, ↑CX-43	20 and 50 (7 days) Dose dependant 50 most effective	I.P. LPS, adult male BALB/c mouse (Yang et al., 2019)
		↓EB leakage, ↑occludin-1, claudin-1, ↑CX43	25, 50, or 100 (7 days)	I.P. LPS, adult male BALB/c mouse (Cheng et al., 2018)

### Anti-oxidative

Oxidative stress is a well-established contributing factor in neurological disorders (Michalska and León, 2020) with high metabolic activity combined with a lack of antioxidant defense capability leaving the brain particularly susceptible. Although citrus polyphenols reportedly demonstrate free radical scavenger capacity *in vitro* (Di Meo et al., 2013), the current literature search unanimously highlighted their ability to stimulate the endogenous antioxidant defense machinery, with superoxide dismutase (SOD), catalase (CAT), glutathione (GSH), glutathione S-transferases (GST), glutathione reductase (GR) and glutathione peroxidase (GPx) activation common across all the citrus polyphenols reviewed (Table 1). Effective dosage ranged from as little as 5 mg/kg up to 300 mg/kg, however, dose dependant effects were established in a number of studies, with greater doses usually eliciting greatest effects (Table 1). Activation coincided with subsequent reduction of reactive oxygen species (ROS) such as hydrogen peroxide (H_2_O_2_), nitric oxide (NO) and other oxidative markers namely malondialdehyde (MDA) and thiobarbituric acid reactive substances (TBARS). Upregulation of the transcription factor NRF2 features in a number of these studies (Bai et al., 2014; Sugumar et al., 2019; Welbat et al., 2020), and is likely central to this polyphenol mediated anti-oxidative system through the activation of the antioxidant response element (Vauzour, 2012). As alluded to, activation of the antioxidant defense machinery was generally consistent across the flavonoids with only a few discrepancies found, mainly confined to naringenin and hesperetin, where occasionally no effect was detected (Chtourou et al., 2014; Kheradmand et al., 2018; Mani et al., 2018). A few studies reported the opposite effect for naringenin with SOD and CAT reduced (Chtourou et al., 2014; Haider et al., 2020). This reduction associated with naringenin, which is believed to be able to cross the BBB (Youdim et al., 2003), may relate back to its free radical scavenging capabilities, or arise as a result of the differing disease models used. In addition to the more common antioxidant machinery influenced, hesperidin and hesperetin upregulated Haem-oxygenase (HO-1) and downregulated the superoxide radical generating enzyme Xanthine Oxidase (XO) respectively (Ashafaq et al., 2014).

### Anti-inflammatory

The immunomodulatory capabilities of citrus polyphenols within the brain are similarly evident, and are in some cases coupled to anti-oxidative mechanisms (e.g., HMGB1/RAGE, Yu et al., 2015). Again, the molecular targets with which the reviewed citrus polyphenols interact appear to be consistent, with reduction of pro-inflammatory cytokines IL-1β, IL-2, IL-6, IFN-γ, and TNF-α, particularly prolific in the literature (Table 1). This is likely mediated through the mitigation of hyperactive immune cells as is suggested by the reduction of GFAP, and NF-κB which governs chemokine and inflammatory mediator transcription (Fu et al., 2019; Zhou et al., 2020). In contrast to evidence on anti-oxidative effects, there was little indication to suggest lack of effect across any of the reviewed flavonoids, supporting the notion that neuro-inflammatory modulation is inherent across all citrus flavonoids. As with anti-oxidative effects, effective dosage had a considerable range of 20–300 mg/kg, with efficacy remaining even at lower doses 25–50 mg/kg. This latter dose equates to 145–290 mg human equivalent dose in a 70 Kg adult, if the animal is a mouse (Nair and Jacob, 2016), which is achievable through consumption of 0.5–0.75 L of orange juice (De Rycker et al., 2020).

### Bioenergetic

Citrus flavonoids appear to ameliorate mitochondrial dysfunction – damage to the mitochondria which may be due to exogenous factors and which can predispose individuals to certain neurodegenerative conditions. As with anti-inflammatory properties, the impact was consistent across the reviewed studies with reported dosages of 25–200 mg/kg resulting in an increase of mitochondrial respiratory chain complexes (I–IV) function and stability. Disturbance of mitochondria function impairs mitochondrial enzyme bioenergetics, reducing ATP production, while simultaneously leading to substantial increases in ROS production thus feeding into the other disease processes.

In addition to mitochondrial function, implications for acetylcholinesterase activity and thus cholinergic transmission were apparent. Generally, citrus flavonoids led to a reduction in acetylcholinesterase activity, accompanied by subsequent increased acetylcholine levels (Ashafaq et al., 2014; Sachdeva et al., 2014; Chtourou et al., 2015; Javed et al., 2015; Khajevand-Khazaei et al., 2018; Ishola et al., 2019; Haider et al., 2020), however the reverse (increased acetylcholinesterase activity) has was also reported (Chtourou et al., 2014; Shagirtha et al., 2017). Again, these discrepancies likely arise as a result of the different models used (Table 1).

### Proteinopathy

Only a limited amount of preclinical evidence exists to support the beneficial effects of citrus on proteinopathies. This primarily relates to Alzheimer’s disease, with reports revealing improvements in Tau phosphorylation (Yang W. et al., 2014; Okuyama et al., 2018; Zhou et al., 2020), and Aβ deposition (Yang W. et al., 2014; Li et al., 2015; Zhou et al., 2020), however no effect has also been reported (Wang et al., 2014). The mechanistic basis for these changes remains relatively weak, although the glycogen synthase kinase 3 beta (GSK3β) may be a plausible candidate (Yang W. et al., 2014; Zhou et al., 2020). A reduction of α-synuclein, the protein attributed to parkinopathy has also been described (Mani et al., 2018), potentially indicating that the effect extends further than Alzheimer’s disease-associated proteins. Higher dosage appears crucial to establish these effects, with <100 mg/kg failing to have any impact. Finally, a report in which hesperetin was administered demonstrated restoration of brain proteolytic enzyme levels (Shagirtha et al., 2017), potentially linking citrus flavonoids to lysosomal degradation processes (Aufschnaiter et al., 2017) (Table 1).

### Anti-apoptotic

Ultimately, neurodegeneration which leads to cognitive impairment can be defined as the progressive loss of neurons. Dysregulation of the above-mentioned processes categorically leads to neuronal cell death. Thus, the modulation achieved through citrus flavonoids demonstrates a switch from a disease state to an overall anti-apoptotic environment and in some cases an additional increase proliferation/neurogenesis. Amongst markers of these effects, the nuclear protein Ki67 and the microtubule-associated protein doublecortin (DCX) were reported in addition to the apoptotic regulating proteins Bax-Bcl2, and ERK-BDNF (Table 1).

### Cognitive Outcomes

Unsurprisingly, given the established effects of flavonoids on key markers of brain health and functionality at molecular and metabolic levels, in studies where cognition was determined, flavonoid supplementation led to improvements in cognitive performance. Cognitive and behavioral assessment further supports/demonstrates the neuroprotective properties associated with citrus flavonoids (Table 2). Anxiolytic and antidepressant actions were particularly prominent across the literature and appeared to be consistent across several disease models, suggesting modulation of a fundamental anxiety and depression related process. Similarly, citrus polyphenol supplementation improved deficits in learning and memory, specifically spatial and recognition memory, which may indicate protection of medial temporal lobe, particularly vulnerable to Alzheimer’s like neurodegenerative diseases. Improvements in motor functions and locomotion were also apparent, and impressively remained even in most severe models such as middle cerebral artery occlusion (Raza et al., 2013), and traumatic brain injury (Chitturi et al., 2019).

**TABLE 2:** Overview of citrus flavonoids on cognitive performance and locomotor activity in preclinical models.

Citrus polyphenol	Behavioral test performance	Reported effective Dose (mg/kg)	Model summary/References
**Hesperidin**	No change locomotor activity (OF), ↓anxiety-like behavior (EPM), ↓depressive symptoms (splash test)	50 (28 days)	Intrastriatal injection 6-OHD, 3–6-month adult male C57BL/6 mouse (Antunes et al., 2020)
	↓Anxiety-like behavior (EPM, OF), ↓Depressive symptoms (FST)	50, 150 (10 weeks) Dose dependent with 150 more effective	I.P injection STZ, adult male Sprague Dawley rat (Zhu et al., 2020)
	↓Anhedonia (SPT), ↓Depressive symptoms (TST and FST), ↓Anxiety-like behavior (OF)	100 and 200 (three times a week for 3 weeks)	Chronic unpredictable mild stress, 6–8-week ICR male mouse (Fu et al., 2019)
	↑Recognition memory (NOL and NOR)	100 (21 days)	IV injection Methotrexate, male Sprague Dawley rat (Naewla et al., 2019)
	↓Anhedonia (SPT), ↓Depressive symptoms (TST, FST, Novelty-suppressed feeding)	50 (14 days)	Concussive head injury, 10-11-week male NMRI mouse (Kosari-Nasab et al., 2018)
	↑Spatial learning and memory (MWM and Y maze)	25, 50, 100 (14 days). Dose dependent with 50 and 100 more effective	L-Methionine treated, male Wistar rat (Hemanth Kumar et al., 2017)
	↓Depressive symptoms (TST and FST)	100 (60 days)	I.P. AlCl3, 10-12-week male albino Wistar rat (Justin Thenmozhi et al., 2017)
	↓Anxiety-like behavior (OF), ↓Depressive symptoms (FST, rearing, grooming, splash test), ↑Spatial learning and memory (MWM), ↑Recognition memory (NOR)	50 (14 days)	Olfactory bulbectomy, male C57BL/6 mouse (Antunes et al., 2016)
	↓Anhedonia (SPT), ↓Depressive symptoms (FST), No change OF	25, 50, 100 (7 days)	I.P. LPS, male ICR mouse (Li M. et al., 2016)
	↓Anhedonia (SPT), ↓Depressive symptoms (FST)	25, 50 (3 weeks)	Chronic mild stress, 5-week male ICR mouse (Li C. F. et al., 2016)
	↑Social behavior (Nest building, resident-intruder assay)	100 (10 days)	Transgenic APP/PS1–21, 5-month male mouse (Li et al., 2015)
	↑Spatial learning and memory (MWM)	100 and 200 (15 days)	Intracerebroventricular injection STZ, 12-month Swiss male albino mouse (Javed et al., 2015)
	↓Depressive symptoms (FST)	25, 50, 100 (21 days)	I.P. STZ, male albino Wistar rat (El-Marasy et al., 2014)
	↓Abnormal thigmotaxis (OF), ↑Recognition memory (NOR), Spatial reference learning and memory (MWM)	50, 100 (16 weeks) Dose dependent with 100 more effective	APPswe/PS1dE9, 3-month male mouse (Wang et al., 2014)
	↓Depressive symptoms (TST), ↓Spatial memory (MWM), No change OF	50 (28 days)	Intracerebroventricular injection 6-OHDA, female C57 BL/6 mouse (Antunes et al., 2014)
**Hesperetin**	↑Spatial learning and memory (MWM and Y-Maze)	50 (5 weeks)	I.P. LPS, 7–8-week male C57BL/6N mouse (Muhammad et al., 2019)
	↑Recognition memory (NOR), ↑Spatial learning and memory (MWM)	5, 50 (3 days)	I.P. scopolamine, male albino mouse (Ishola et al., 2019)
	↑Recognition memory (NOR), ↑Associative memory (PAT)	10, 20 (3 weeks)	I.P. STZ male Wistar rat (Kheradmand et al., 2018)
**Naringenin**	↑Spatial learning and memory (MWM and Barnes maze)	250-300 (10 weeks)	High-fat diet-fed, 10-month SAMP8 male mouse (Zhou et al., 2020)
	↑Spatial learning and memory (MWM), ↑Recognition memory (NOR), ↑Associative memory (PAT), ↑Working memory (EPM), ↑Social behavior, ↓Depressive symptoms (FST)	50 (14 days)	I.P. AlCl_3_ + D-gal injection, young albino Wistar rat (Haider et al., 2020)
	↑Spatial learning and memory (MWM), ↑Recognition memory (NOR)	100 (15 days)	Oral MeHg, 5-week male Swiss albino mouse (Krishna Chandran et al., 2019)
	↓Anxiety-like behavior (OF), ↓Depressive symptoms (FST), ↑Spatial learning and memory (MWM)	50 (4 weeks)	Chronic unpredictable mild stress, adult male Wistar rat (Tayyab et al., 2019)
	↓Anhedonia (SPT), ↓Depressive symptoms (TST and FST)	25, 50, 100 (5 days) Dose dependent with 50 and 100 more effective	Bilateral olfactory bulbectomy, 3-month BALB/c male mouse (Bansal et al., 2018)
	↑Spatial learning and memory (Y-maze, ↑Recognition memory (NOR), ↑Associative memory (PAT)	50, 100 (7 days)	I.P. LPS male albino Wistar rat (Khajevand-Khazaei et al., 2018)
	↑Motor Function (beam walk, vertical grid, horizontal grid)	25, 50, 100 (5 days)	I.P. injection MPTP, male C57BL/6J mouse (Mani et al., 2018)
	↑Spatial learning and memory (MWM)	50 (16 days)	Young adult male albino Wistar rat (Liaquat et al., 2018)
	↑Spatial learning and memory (Y-maze, RAM), ↑Associative memory (PAT)	100 (single dose)	Dorsal hippocampal Aβ injection, adult male Wistar rat (Ghofrani et al., 2015)
	↓Anhedonia (SPT), ↓Anxiety (Novelty-suppressed feeding test)	10, 20 (3 weeks)	Chronic unpredictable mild stress, 5-week male ICR mouse (Yi et al., 2014)
	↑Spatial learning and memory (MWM)	25, 50, 100 (21 days)	Intracerebroventricular injection, STZ adult male Sprague Dawley rat (Yang W. et al., 2014)
	↑Motor Function (Rota rod, Grip test, Adhesive-removal test)	50 (21 days)	Middle cerebral artery occlusion, 16-week male Wistar rat (Raza et al., 2013)
**Naringin**	↑Locomotor activity (Rotarod, Beam-crossing, footprint analyses), ↓Depressive symptoms (Grooming)	40 and 80↑(28 days)	Intrastriatal injection quinolinic acid, adult male Sprague-Dawley rat (Cui et al., 2018)
	↑Motor/locomotor activity (Horizontal Bar, Spontaneous Motility, Forelimb Flexion, Righting Reflex, Actophotometer, Rotarod, Paw withdrawal) ↓Depressive symptoms (FST) ↑Spatial learning and memory (MWM)	20 and 40 (28 days) Dose dependent 40 most effective	Intastriatal injection collagenase, adult female Wistar rat (Singh et al., 2017)
	↓Motor abnormalities (Hind limb function test, Grip strength test, Print length analysis)	80 (14 days)	I.P. 3-nitropropionic acid, male Wistar rat (Gopinath and Sudhandiran, 2016)
	↑Spatial learning and memory (MWM), ↑Recognition memory (NOR)	100 (20 weeks)	High-fat diet induced cognitive decline, male C57BL/6 mouse (Wang et al., 2015b)
	↓Anxiety-like behavior (OF, elevated T maze)	25, 50, 100 (5 weeks) Dose dependent with 100 most effective	Cisplatin exposed, middle aged Wistar rat (Chtourou et al., 2015)
	↑Spatial learning and memory (MWM) ↑Working memory (EPM)	50, 100, and 200 (21 days)	Intracerebroventricular STZ, adult male Wistar rats (Sachdeva et al., 2014)
**Kaempferol**	↑Sensorimotor behaviors (Whisker stimulation-induced motor response, Forelimb usage test)	1 (three times: 1, 24, 48 h)	Traumatic Brain Injury, 23–24-day male Sprague-Dawley rat (Chitturi et al., 2019)
	↑Spatial learning and memory (Y-maze), ↑Recognition memory (NOR)	21 (14 days)	I.P. Chlorpyrifos daily for 14 days, 8-week male albino Wistar rat (Hussein et al., 2018)
	↑Spatial learning and memory (MWM)	10 (21 days)	Intracerebroventricular injection STZ, OVX female Wistar rats (Kouhestani et al., 2018)

## Human Evidence for Citrus Polyphenols and Brain Health

Whilst a wealth of pre-clinical (reviewed above) and *in vitro* data exploring neurocognitive outcomes in relation to citrus flavonoids is currently available, the same cannot be said for human trials which remain considerably limited in number. In our literature search, we identified 10 human studies (five observational; five interventions) assessing the effects of citrus flavonoids on brain health and cognition in healthy adults, or in addition to other co-morbidities including depression, dementia, schizophrenia, and stroke.

### Studies on Healthy Adults

The benefits of citrus fruit consumption in the context of healthy aging were highlighted by a cross-sectional study involving 2031 elderly (aged 70–74 years) Norwegian individuals which explored the impact of different plant foods on cognitive performance (Nurk et al., 2010). Study participants underwent extensive cognitive testing in addition to completing comprehensive food frequency questionnaires. After adjustment for multiple testing, citrus fruits had the strongest association with cognitive test performance. Kendrick object learning, trial making, digit symbol and block design tasks all showed statistically significant improvements suggestive of better episodic memory, executive function, perceptual speed, and visuospatial skills (Table 3).

**TABLE 3:** Overview of intervention studies on citrus fruits in brain health and disease in humans.

Study	Study design	Participants who completed study	Disease/state	Intervention or variable of interest	Duration
Lamport et al., 2016	Single blind randomized trial	40 healthy adults (18–30 years)	N/A (Healthy adults)	High flavanone drink (70.5 mg)	Acute
Alharbi et al., 2016	Double blind randomized trial	24 healthy males (30-65 years)	N/A (Healthy adults)	Flavonoid rich orange juice (272 mg)	Acute
Kean et al., 2015	Double blind randomized control trial	37 healthy adults (60–81 years)	N/A (Healthy adults)	High flavanone 100% orange juice (305 mg)	8 weeks
Park et al., 2020	Randomized single blind study	40 adults (20–30 years)	Depressive symptoms and microbiota	Flavonoid rich orange juice (600 mg)	8 weeks
Chang et al., 2016	Prospective cohort study	82,643 females (36–55 and 53–80 years)	Depression	Total dietary flavonoid intake (including flavanones)	10 year follow up
Zhang et al., 2017	Retrospective cohort study	13,373 adults (>65 years)	Dementia	Daily citrus intake	5.7 year follow up
Bruno et al., 2017	Open label pilot study	20 adults (age n/a)	Cognitive dysfunction in Schizophrenia	Bergamot polyphenolic fraction (1000 mg/day)	8 weeks
Nurk et al., 2010	Cross-sectional study	2031 Elderly adults (70–74 years)	Cognitive decline	Different plant foods (including citrus intake)	N/A
Goetz et al., 2016	Biracial prospective study	20,024 participants (>45 years)	Stroke incident	Different flavonoid intake (including flavanones)	6.5 years follow up
Cassidy et al., 2012	Prospective cohort study	69,622 females (30–55 years)	Stroke incident	Dietary flavonoid intake (including flavanones)	14 years follow up

Following on from these observations, Kean et al. (2015), examined the chronic consumption of flavanone-rich orange juice in relation to cognition in 37 healthy volunteers (60–81 years). The study was a double-blind RCT with a crossover design and involved consumption of a 500 mL high flavanone (daily serving of 305 mg – 549 mg hesperidin/L and 60 mg narirutin/L) orange juice daily for 8 weeks. In support of the cross-sectional study mentioned above, global cognition increased in response to chronic consumption of the flavanone-rich orange juice relative to control. In addition, high flavanone intake significantly improved recall and nominally increased executive function, although *post hoc* analysis found this to be non-significant (*p* = 0.06). These effects were independent of mood and blood pressure which both remained unchanged (Table 3).

Acute neurological responses to citrus flavonoids have similarly been investigated (Alharbi et al., 2016; Lamport et al., 2016). Firstly, in a randomized, double-blind, placebo-controlled, crossover trial, Alharbi et al. (2016) explored the cognitive benefits associated with flavonoid-rich orange juice (272 mg – 220.46 mg hesperidin, 34.54 mg narirutin, 17.14 mg other flavonoids) at 2 and 6 h post-consumption in 24 healthy middle-aged adults (30–65 years). From the cognitive battery performed, flavonoid rich orange juice consumption led to higher performance in Simple Finger Tapping (measure of psychomotor speed) and Continuous Performance Task (measure of attention and more broadly executive function) at 2 and 6 h respectively. A non-significant trend for higher global cognitive performance (all tests combined) was also observed, as well as an increase in subjective alertness. Interestingly, significant improvements observed in cognition and subjective alertness at 6 h coincide with an anticipated peak in flavanone metabolites at 5–7 h (Manach et al., 2003), although this was not physically measured.

As with the previous study, Lamport et al. (2016) assessed acute neurological response, this time to a commercially available high flavanone beverage (HF, 70.5 mg – 42.15 mg hesperidin, 17.25 mg naringin, 6.75 mg narirutin, 4.3 mg caffeic acid) and utilizing an additional measure of cerebral blood flow (CBF). The study was single blind, randomized, cross over, by design involving healthy adult volunteers (aged 18–30 years). Participants underwent either cognitive testing 2 h post consumption (*n* = 28) or completed an fMRI assessment of CBF 2 and 5 h post consumption (*n* = 16). High flavanone beverage intake significantly increased cerebral perfusion in the inferior frontal and middle right frontal gyrus in the right hemisphere at 2 h. Similarly, at 2 h, improvement in digit symbol substitution test (a measure of executive function) was seen, correlating with the increased regional perfusion of the inferior frontal gyrus, known to be involved in executive function (Aron et al., 2004). Despite the extensive cognitive battery, no additional effects were found. However, as addressed by the authors, this study had a number of limitations. In particular, the fact MRI and cognitive test were performed on separate individuals, and that cognitive tests were only performed at 2 h limits comparative potential. Additionally, as stated by the authors, the fact that the participants were generally young and highly educated may have limited the response given they were likely to be optimally functioning (Table 3).

### Studies on Depression

Depression is a complex mood disorder which can often be challenging to treat effectively. Preclinical studies have reported anti-depressant effects of flavonoids, usually attributable to their antioxidant and anti-inflammatory characteristics, and inhibition of monoamine oxidases (Hritcu et al., 2017). An important serological marker identified in depression is BDNF (involved in processes within the central nervous system) which has been observed to be significantly lower in patients with major depressive disorder compared to non-depressed control groups, and subsequently recovered antidepressant users (Chen et al., 2001). Interestingly, BDNF is often increased in response to flavonoid consumption (Neshatdoust et al., 2016).

In a prospective cohort study following 82,643 women with no previous diagnosis of depression from the Nurses’ Health Study (NHS; aged 53–80 years) and Nurses’ Health Study II (NHSII; aged 36–55 years) throughout a 10-year period, an inverse association between incident depression and citrus intake, with greatest flavanone intake (>64.2 mg/day) resulting in a significant 10% reduction in incident depression risk was observed. Furthermore, citrus fruit and juice intake of >2 servings/day had HR of 0.82 (Chang et al., 2016).

These anti-depressant effects of citrus flavonoids were recently put to the test by Park et al. (2020), in a single blind, randomized control study examining the effects of daily 380 ml flavonoid rich (FR; 600 ± 5.4 mg flavonoid content) orange juice consumption on depressive symptoms and gut microbiota for an 8-week period in young adults (aged 20–30 years). The results in relation to depression were by no means clear cut, with no apparent significant differences between high and low flavonoid groups after 8 weeks. However, compared with baseline the results suggested potential improvement in BDNF, and to some extent to the Center for Epidemiological Studies Depression Scale (CES-D), a psychiatric screening tool used to detect pre-existing mental disorders, although both treatments appeared to improve upon baseline CES-D scores (Table 3).

### Studies on Dementia

Despite the preclinical evidence backing the beneficial effect of citrus flavonoids in models of Alzheimer’s and Parkinson’s diseases, etc. there remains very limited evaluation at the human level.

In a retrospective cohort study (Zhang et al., 2017), examined the association between daily citrus intake and dementia incidence in 13,373 participants (age ≥ 65 years). FFQs in combination with the Japanese Long-term Care Insurance database were used over a 5.7 years follow up period. Overall, an inverse dose-response relationship was established between weekly citrus fruit intake and incident dementia, with hazard ratios for citrus fruit consumption 3–4 times/week and every day 0.92 (95% CI 0.80, 1.07) and 0.77 (95% CI 0.73, 1.01) respectively. As dementia represents a wide range of neurodegenerative diseases, lack of further classification limits the extent to which we can attribute these effects to specific neurodegenerative diseases and should be considered in future studies (Table 3).

### Studies on Schizophrenia

Schizophrenia is a complex psychotic condition affecting cognition, the cognitive hallmark of schizophrenia being poor learning and retention of verbal information (Bowie and Harvey, 2006). In an open label pilot study, 20 outpatients diagnosed with schizophrenia receiving second generation antipsychotic medication consumed a flavanone-rich bergamot polyphenolic fraction (BPF) daily at a dose of 1000 mg/day for 8 weeks. Daily BPF consumption significantly improved Wisconsin Card Sorting Test “perseverative errors” and semantic fluency test. With a trend for other cognitive variables evident (Bruno et al., 2017) (Table 3).

### Studies on Stroke and Stroke Risk Factors

The protective effects of citrus flavonoids in cerebrovascular disease are well documented (Testai et al., 2017; Mahmoud et al., 2019) and appears to extend to stroke incidence. A prospective cohort study, assessing the association of dietary flavonoid intake toward stroke risk, followed 69,622 women (30–55 years) from the NHS Study throughout a 14-year period (Cassidy et al., 2012). Although flavonoid intake was not inversely associated with stroke risk, women with greatest intake of flavanones > 62.95 mg/day had a relative risk for experiencing an ischemic stroke of 0.81 compared to lowest intake < 13.72 mg/day. Additionally, an inverse trend was established between citrus fruit/juice intake and risk of ischemic stroke. These apparently protective effects did not influence haemorrhagic strokes (Scheffers et al., 2018; D’Elia et al., 2020; Zurbau et al., 2020).

A recent prospective cohort study (Goetz et al., 2016), utilizing ‘The Reasons for Geographic and Racial Differences in Stroke’ (REGARDS) database, assessed whether any association exists between flavonoid intake and incident ischemic stroke in a biracial cohort. 20,024 participants (>45 years) of whom flavonoid consumption was determined from FFQ, were followed for 6.5 years. After multivariable adjustment, the highest flavanone intake > 48 mg/day was inversely associated with incidences of ischemic stroke compared to lowest flavanone intake < 3.9 mg/day. With citrus fruit/juice consumption displaying similarly reduced risk (HR of 0.69). In agreement with the above-mentioned study of total flavonoids and other flavonoid subclasses did not show any statistically significant association with incident ischemic stroke, clearly showcasing the importance of the flavanone subclass (Table 3).

Of particular relevance to the mitigation of stroke risk, and also neurodegenerative conditions, it is worth briefly mentioning the influence of citrus flavonoid intake on blood pressure, and vascular functions. RCTs in which flavonoids were administered in the form of orange juice have established significant reductions in blood pressure (Morand et al., 2011; Valls et al., 2020), pulse pressure (Valls et al., 2020), while also improving flow-mediated dilation (Li et al., 2020).

## Future Perspectives and Conclusion

The preclinical literature search distinguished fundamental mechanisms central to citrus flavonoids, with protective effects linked with anti-oxidative and anti-inflammatory action particularly well established. Yet, there are a number of areas requiring further investigation. First, the overwhelming majority of studies to date involve relatively young male animals making it difficult to establish whether or not the observable effects are sex or age specific. Nor do we have an understanding of modulation by either factor. Second, the models employed tend to lean toward the severe side of the disease spectrum, thus translation to a healthy aging context, or milder conditions, remains to be fully determined, although some initial work provides supportive results (Liaquat et al., 2018). Third, despite recent attention on the relationship between gut microbiota and polyphenols, and the apparent ability of citrus flavonoids to mitigate LPS associated neuroinflammation/NDG, to our knowledge there are no studies which have explored the role of the microbiota in combination with citrus flavonoids in the context of brain health and disease. Finally, as can be seen in Table 1, the impact of citrus flavonoids upon BBB integrity/function appears to have emerged over the last couple of years (Cheng et al., 2018; Li et al., 2019; Yang et al., 2019; Lee et al., 2020), particularly for kaempferol, and therefore should be examined in further studies, especially as BBB integrity has similarly been associated with gut microbiota (Braniste et al., 2014).

From a human perspective, there is an obvious lack of human clinical studies which needs to be addressed if a robust assessment of therapeutic potential is to be made. Similarly, very few human studies have followed up on the mechanistic insights established in the preclinical setting. Future human studies should take note of the limitations arising from other human studies in which nutraceuticals were assessed in the context of brain health and disease, for example ensuring optimal participant targeting (in demographics where significant change is most likely to occur), dosage, timing, and duration of treatment for measurable effects to be established.

The complex mixtures of polyphenols present in citrus fruits and juices and their bioactive nuances likely convey greater benefit than one purified compound, accumulatively acting upon multiple targets, and producing synergistic effects. Given the multifactorial nature of neurodegenerative diseases, one would speculate that this complex form would therefore offer greater efficacy, but this is yet to be fully determined.

In conclusion, although significant work remains to fully establish the benefits of citrus polyphenols in brain health and disease, the accumulating *in vitro* and preclinical data combined with the support of steadily emerging human studies indicates future potential.

## Author Contributions

MP, MMM, and DV wrote the manuscript. MP, MMM, EC, MM, and DV contributed to the literature search and edited the manuscript. All the authors contributed to the article and approved the submitted version.

## Conflict of Interest

MP and DV received unrestricted honorariums from the Fruit Juice Science Centre. The article reflects the views of the authors alone, and the funding source had no role in the preparation or submission of the manuscript. The remaining authors declare that the research was conducted in the absence of any commercial or financial relationships that could be construed as a potential conflict of interest. The handling editor declared a past co-authorship with one of the authors DV.
